# Wireless Temperature Sensor Based on a Nematic Liquid Crystal Cell as Variable Capacitance

**DOI:** 10.3390/s18103436

**Published:** 2018-10-12

**Authors:** Juan Carlos Torres, Braulio García-Cámara, Isabel Pérez, Virginia Urruchi, José Manuel Sánchez-Pena

**Affiliations:** Displays and Photonic Applications Group (GDAF-UC3M), Electronic Technology Department, Carlos III University of Madrid, Av de la Universidad 30, E28911 Leganés, Spain; brgarcia@ing.uc3m.es (B.G.-C.); isaper@ing.uc3m.es (I.P.); vurruchi@ing.uc3m.es (V.U.); jmpena@ing.uc3m.es (J.M.S.-P.)

**Keywords:** nematic liquid crystal, impedance analysis, temperature dependence, equivalent circuit, capacitive sensor, simulation

## Abstract

Wireless communication is growing quickly and now allows technologies like the Internet of Things (IoT). It is included in many smart sensors helping to reduce the installation and system costs. These sensors increase flexibility, simplify deployment and address a new set of applications that was previously impossible with a wired approach. In this work, a wireless temperature sensor based on a nematic liquid crystal as variable capacitance is proposed as a proof of concept for potential wearable applications. Performance analysis of the wireless temperature sensor has been carried out and a simple equivalent circuit has been proposed. Sensor prototype has been successfully fabricated and demonstrated as the beginning of new biomedical sensors.

## 1. Introduction

The large variety of commercial temperature sensors shows the importance of measuring this parameter. Temperature changes are crucial in biological processes [1], manufacturing techniques [2] or biomedical applications [3], among others. For this reason, commercial sensors should be able to work in diversified operation conditions, as well as different temperature ranges. This is possible by using different physical and/or chemical processes and materials as the basis of these sensors. Thermocouplers use the thermo-electric effect in a metal-metal junction [4], while a resistor temperature detector (RTD) is based on the temperature-dependent resistivity of a metallic wire [5]. An optical-fibre temperature sensor [6] uses the spectral emission of an object as a function of its temperature following the black-body radiation principle. Other examples are thermistors, integrated circuit (IC) temperature sensors and so forth. These are just some of the long list of commercial devices.

Liquid crystals (LCs) can also be used in the design of temperature sensors. These media are composed of nanometric elongated organic molecules producing an orientational order [7]. In addition, LCs present a high thermo-optic coefficient [8,9], making them also the base of high-sensitivity temperature sensors [10]. Not only the LC material but also geometrical and design parameters of the device, such as the thickness of the cell or the initial alignment of the LC, are important to determine the sensitivity of the sensor. For this reason, an electrical model has been developed in the past [11] helping in the analysis and integration of this kind of sensors. While LC-based temperature sensors require that the LC is in the anisotropic phase (e.g., nematic) which limits the operation range up the clearing point [7], there are several formulations of LCs offering a very large range of temperatures, from 35 °C of the well-known 5CB to higher than 170 °C in other kind of LCs [12].

Although commercial temperature sensors with physical connections have a price advantage, the sensor industry is dynamic and customers are seeking more flexible and reliable new applications that are impossible with a wired approach. Additionally, the recent advances in the Internet of the Things (IoT) are demanding novel sensors with new architectures [13]. Temperature sensors are also strongly demanded in the e-medicine [14]. In both cases, one of the most important characteristics is the wireless connection of these sensors. This also allows that sensors can work in harsh environments [15] or may reduce the connection and power requirements in integrated complex systems, like on-board sensors in airplanes or wearable biomedical applications.

Several works in the bibliography use resonant circuits to perform sensors with wireless connection [16]. Pressure [17], humidity [18] and even temperature sensors [15,19] implementing wireless communications have been already developed. However, for the best of our knowledge, the use of a nematic LC cell as a wireless temperature sensor has not been demonstrated before. Resonant circuits are one of the most extended ways to point-to-point communications without a physical connection. A LC cell has a capacitive behaviour, which depends on the temperature, thus an inductive-capacitive resonant circuit, including the LC cell, is an excellent alternative.

In this work, a wireless temperature sensor using the capacitance of a LC cell as sensing parameter is proposed. In addition, this sensor has been included in an inductive-capacitive resonant circuit to ensure wireless connection. The electric response of the complete system has been analysed and an equivalent circuit model has been proposed. This makes possible to optimize the device taking conscience of the influence of each parameter. Finally, the detailed theory to describe the behaviour of the circuit has been validated through simulations and experimental measurements. This prototype is conceived as the germ of a wearable temperature sensor for biomedical monitoring.

## 2. Design of the Sensor

**Figure 1:**
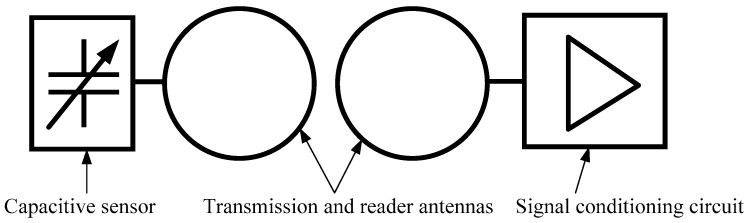
Diagram of the proposed wireless temperature sensor.

The proposed wireless temperature sensor consists of a capacitive sensing element connected to an inductor forming a resonant circuit. A remote reader antenna sending out an oscillating magnetic field (Figure 1) electrically excites this. Any temperature change produces a variation of the capacitance of the sensing element. The reader can detect this variation by monitoring the impedance because capacitance changes produce resonant frequency shifts [20].

The novelty of this work lies on the capacitive element. This is performed using a liquid crystal cell. In particular, the chosen liquid crystal is a nematic one (NLC 6290, Merck, Darmstadt, Germany). This NLC has a positive dielectric anisotropy (Δ*ε*) around 10 at low frequencies and a clearing point of 104 °C. NLC is sandwiched in a cell composed of two glass plates with an area *A* = 1 cm^2^, both covered with a conductive film. In this case, the film is made of ITO (indium-tin-oxide). This structure behaves as a flat parallel-plate capacitor, in which NLC acts as dielectric.

**Figure 2:**
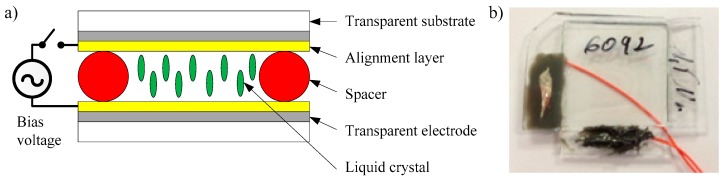
(**a**) Schematic illustration of homeotropic alignment in a nematic liquid crystal cell with no applied external voltage (V_BIAS_ = 0 V). (**b**) Picture of an experimental NLC cell used in the wireless temperature sensor.

The capacitance of this kind of capacitors can be expressed as:

(1) $$C=\left(\varepsilon_{0}\cdot \varepsilon^{\prime }A\right)/d$$

where *ε*_0_ is the dielectric permittivity of the vacuum, *ε*′ is the effective relative permittivity of the dielectric, *A* is the effective area of the electrodes and *d* is the cell gap. Therefore, capacitance strongly depends on the gap between both plates. In this work, this gap is fixed to 1.5 µm using spacers located along the perimeter of the plate area, as it is shown in Figure 2. This particular value has been selected because we experimentally observed, in a previous work [11], that these geometrical conditions provides a high temperature sensitivity. In that previous work, we also obtained, from experimental measurements and using a cell with a thickness of 6.3 µm, that the dielectric constant of the NLC 6290 is (${\varepsilon^{\prime }}_{∥},\;{\varepsilon^{\prime \prime }}_{∥}$) = (13.86, 0.78) and (${\varepsilon^{\prime }}_{⊥},\;{\varepsilon^{\prime \prime }}_{⊥}$) = (4.05, 0.02) at 10 kHz. These values may differ from those of the bulk material due to voltage shielding effect from alignment layers, as was described in Reference [21]. This should be taken into account for further improved designs. In particular, we will show the differences between these values and those of the present work below.

In the same way, the alignment of the LC into the cell is homeotropic [22] instead of homogenous because of its larger sensitivity to temperature changes [10]. This is due to the different temperature dependence of the two effective dielectric permittivities (${\varepsilon^{\prime }}_{⊥},$ and ${\varepsilon^{\prime }}_{∥}$) of the LC in its anisotropic phase (liquid-crystal phase). A convenient alignment of the molecules inside the manufactured device provides the dominance of one of them or a combination of both. Because the stronger temperature dependence of the relative permittivity in the direction parallel to the long molecular axis, a homeotropic alignment has been used in the device to increase the sensor sensitivity to temperature changes. Once the material changes to the liquid phase (isotropic phase), the NLC behaves like a conventional liquid with a single effective dielectric permittivity that remains almost constant with the temperature. In this work, the operation temperature range has been constrained between 10 °C to 80 °C, according with a biomedical application. This is a narrow but practical range for a large variety of other applications and it allows that the maximum temperature is well below the clearing point of NLC, therefore, the material remains in the anisotropic phase. This range can be extended close to the clearing point, or even more by choosing other LCs.

**Figure 3:**
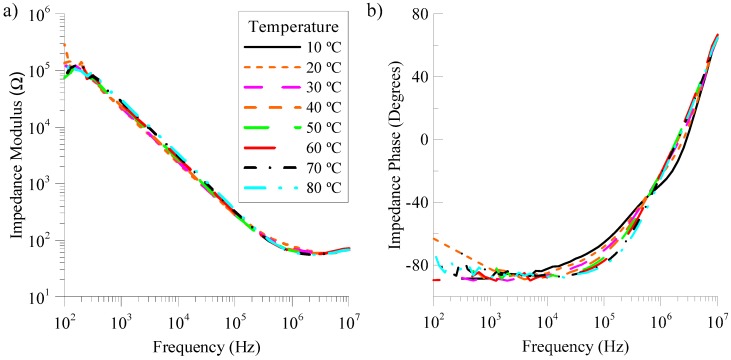
Magnitude (**a**) and phase (**b**) of the impedance of a nematic liquid crystal cell (NCL) as a function of the frequency of the external voltage signal for several temperatures of the environment.

Experimental samples of NLC have been characterized to determine their capacitance at different temperatures. In this sense, the complex impedance has been measured with an impedance analyser (SOLARTRON 1260) using a sinusoidal voltage signal of 100 mV_RMS_ and a frequency sweeping in a range from 100 Hz to 10 MHz. Temperature control has been made using a programmable environmental chamber (DICOMETAL CCK-40/180). In this experiment, temperature takes values from 10 °C to 80 °C with steps of 10 °C. Results of the impedance, both magnitude and phase, are displayed in Figure 3. The frequency range at which the impedance phase is −90° corresponds to a pure capacitive behaviour, in which we are interested. In this case, this frequency range spreads from 1 kHz to 20 kHz, in the considered temperature range. The low limit of the range is restricted by the appearance of certain anomalies in experimental measurements of the impedance, both modulus and phase, at low frequencies (hundreds of Hz). These are probably related with the ionic effects of the NLC [23]. Again, this range can be tuned by using a different LC material. The present work is shown as proof-of-concept. For this reason, an operative prototype is characterized, although it is not optimum.

## 3. Methods

An interesting way to control and fully understand the behaviour of the proposed sensor is by means of an electric equivalent circuit (EEC). This provides a simple electric circuit with discrete elements. This section is devoted to proposing an EEC of both the sensing element and the complete sensor, including the wireless connection.

### 3.1. Electric Equivalent Circuit of the Sensing Elements

**Figure 4:**
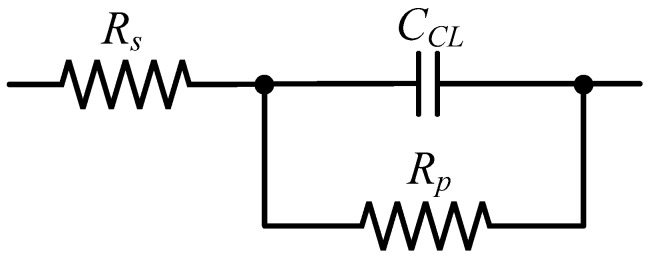
Electric equivalent circuit of NLC device.

The equivalent circuit of the LC cell was deduced using the previous complex impedance measurements. Figure 4 shows that the NLC samples has an EEC consisting on an ideal capacitor with two resistors: one in series, *R_s_* and one in parallel, *R_p_*. This is the typical representation of any LC cell [24] in a wide range of frequencies. The capacitive behaviour can be clearly observed from Figure 3. In particular, this capacitive behaviour is dominant in the frequency range in which the phase is constant and equal to −90° (1 kHz–20 kHz). The resistive effect is intrinsic to any real circuit. In this case, we identify two main sources: the resistance due to the connecting wires (*R_s_*) and that of the conductive layers (*R_p_*). Their value can be estimated from experimental measurements.

In the high-frequency range (~MHz), the behaviour of the capacitor (*C_CL_*) becomes comparable to a short-circuit because the small value of the impedance magnitude, as it can be seen in Figure 3a. Then, the impedance of the device can be considered equal to only *R_s_*. On the other hand, at low frequencies, the capacitor presents a large impedance. Consequently, it behaves like an open-circuit. Under this assumption, the electric model can be simplified to the effect of *R_s_* and *R_p_* in series.

**Figure 5:**
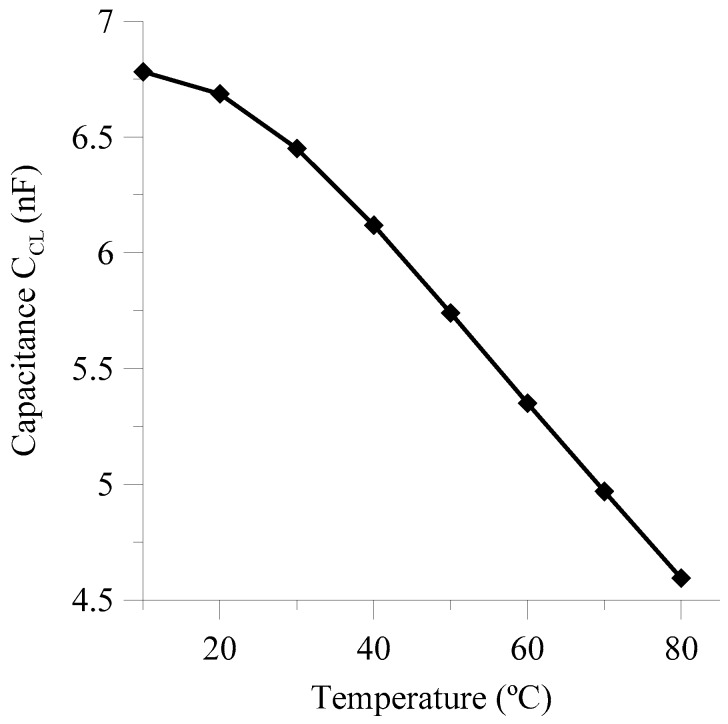
Variation of NLC cell capacitance as a function of temperature at a frequency of 10 kHz.

From the previous results, the effective capacitance of the NLC cell as a function of the temperature was deduced and plotted in Figure 5 at a frequency of 10 kHz. This curve shows a decreasing monotonous tendency of NLC cell capacitance with a slope of −30 pF/°C. From this capacitance and using Equation (1), we can estimate the value of ${\varepsilon^{\prime }}_{∥}$~11.3 at room temperature. It can be seen that the thin thickness of the considered cell strongly influences on these value, as commented in Reference [21].

### 3.2. Electric Equivalent Circuit of the Whole Sensor

**Figure 6:**
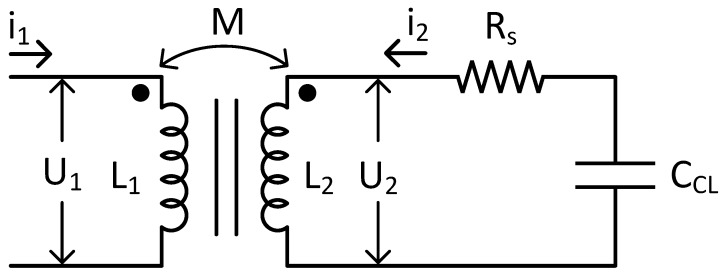
Electric model of the wireless temperature sensor considering a real transformer.

**Figure 7:**
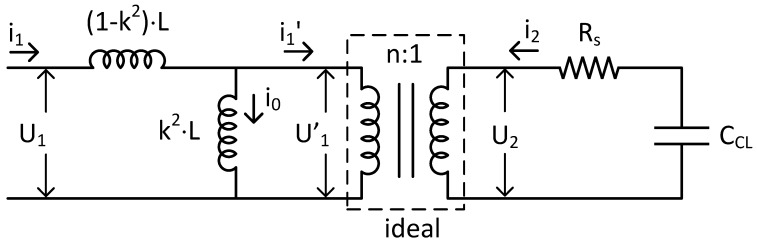
Electric equivalent circuit of wireless temperature sensor with a modelled transformer.

**Figure 8:**
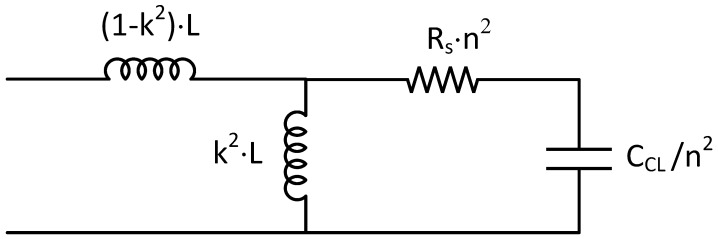
Simplified electric equivalent circuit of wireless temperature sensor.

As before, a complete analysis of the electric response of the circuit, including also the antenna, has been performed. Figure 6, Figure 7 and Figure 8 show the schemes of the evolution from the real to a total electric equivalent circuit. Figure 6 corresponds to the real circuit. In this one, the sensor’s inductor and the reader’s antenna has been modelled as a real transformer. In a real transformer, there is a mutual inductance *M* simulating the inductive link. *M* could express the coupling. *U*_1_, *U*_2_, *i*_1_ and *i*_2_ are respectively the primary and secondary voltages and the primary and secondary current. Taking advantage of the previous model, in the considered frequency range, the NLC sample is equivalent to a resistor and capacitor in series. This means that the sensor is working in the frequency range in which the NLC cell has a predominant capacitive behaviour.

An evolution of the previous circuit is that considering the detail of the transformer. Consequently, Figure 7 includes the equivalent circuit of the proposed transformer. This is composed of an ideal transformer and two different inductances. The coupling coefficient, *k*, gives idea about the linking capability between the primary and secondary windings (*L*_1_ and *L*_2_). This is one of the dominating factors in the wireless communication determining the readout distance. Additionally, *n* is the turns ratio of the ideal transformer. The coupling coefficient *k* and the inductances of the windings of the real transformer depend on this value. The values of model parameters (*k*, *n*, *L*) have been obtained by matching with real circuit parameters (*L*_1_, *L*_2_ and *M*). A detailed explanation is included below.

The primary and the secondary voltages in the real circuit (Figure 6) can be written as a function of Laplace variable (*s*) as follows:

(2) $$U_{1}=L_{1}\cdot s\cdot i_{1}+M\cdot s\cdot i_{2}$$

(3) $$U_{2}=M\cdot s\cdot i_{1}+L_{2}\cdot s\cdot i_{2}$$

These voltages in the circuit with the model of transformer (Figure 7) are:

(4) $$U_{1}=\left(1-k^{2}\right)\cdot L\cdot s\cdot i_{1}+k^{2}\cdot L\cdot s\cdot i_{0}\to U_{1}=L\cdot s\cdot i_{1}+k^{2}\cdot L\cdot s\cdot i_{2}/n$$

(5) $$U_{2}=U_{1}^{\prime }/n=\left(k^{2}/n\right)\cdot L\cdot s\cdot i_{0}\to U_{2}=\left(k^{2}/n\right)\cdot L\cdot s\cdot i_{1}+{\left(k/n\right)}^{2}\cdot L\cdot s\cdot i_{2}$$

By matching these expressions, the relationships between the effective parameters of the modelled transformer and the real circuit are

(6) $$M=k^{2}\cdot L/n$$

(7) $$L=L_{1}$$

(8) $$k=M/\sqrt{L_{1}\cdot L_{2}}$$

Using these relationships, the simplified electric circuit is shown in Figure 8. This simple circuit is able to reproduce the electric behaviour of the considered sensor, including the wireless connection.

Using the LC resonator model in Figure 8, the resonant frequency (*f*) can be easily obtained as follows:

(9) $$f=1/2\cdot \pi \cdot \sqrt{k^{2}\cdot L_{1}\cdot C_{CL}/n^{2}}$$

## 4. Results and Discussion

**Figure 9:**
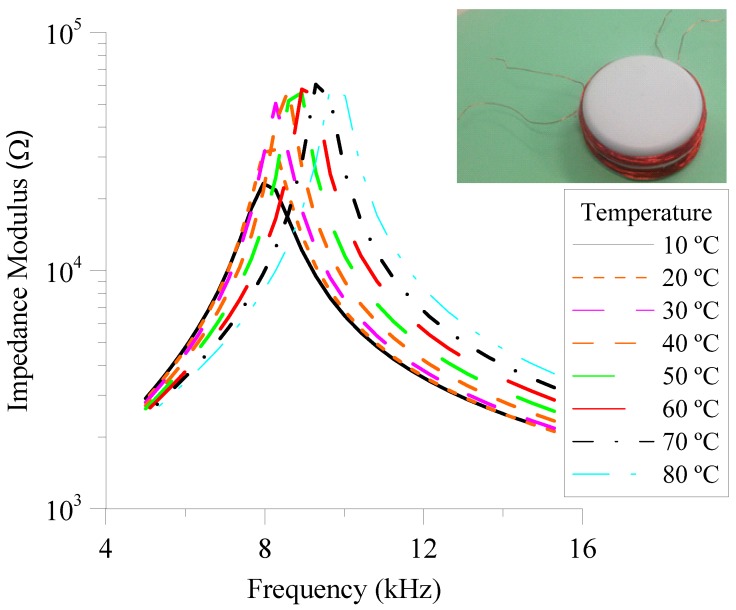
Impedance magnitude of the LC circuit as a function of the frequency for several temperatures. Inset: Prototype of the emitter and receiver antennas.

To provide a wireless communication, two-spiral inductors have been fabricated. These antennas have a radius of 6 cm and 332 turns of a wire with a diameter of 0.1 mm (see inset of Figure 9). These characteristics produce an effective inductance of 55.888 mH. In this case, both antennas, the emitter and the receiver, have been considered equal for simplicity. The previous NLC device connected to the transmission antenna forming a resonant circuit provides a resonant frequency of 10 kHz, approximately. In particular, Figure 9 shows the RF response of the complete circuit over the full range of temperatures considered (10 °C–80 °C). With a nominal capacitance value of 6.45 nF at T = 30 °C, the resonant frequency is 8274 Hz. As the temperature changes, the dielectric properties of the LC material change, resulting in variation of the electric capacitance and a shift of the resonant frequency.

**Figure 10:**
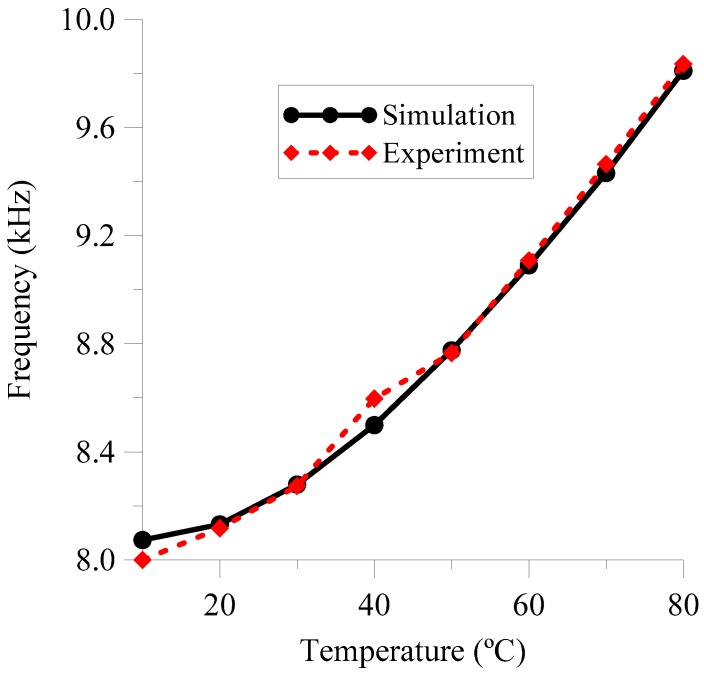
Evolution of the resonance frequency as a function of the temperature. Both experimental measurements and theoretical results (using the previous EEC model) have been included.

Figure 10 shows the resonant frequency as a function of the external temperature. This figure shows a nonlinear response of the sensor with a sensitivity around to 24.8 Hz/°C and a nonlinearity of 18.35%. In addition, the proposed EEC has been tested. In the experiment both antennas are equal and close one to the other, with an inductance of L = 55.888 mH. For this reason, both *k* and *n* are equal to 1 in the circuit of Figure 8. By means of the characterization of the NLC, the capacitance at different temperatures can be obtained. Using these values, the simulated resonant frequency was calculated and plotted as a function of the temperature in Figure 10. As it can be seen, there is a relevant agreement between the experiment and the proposed model. This provides a powerful tool to analyse temperature sensors based on LC cells.

## 5. Conclusions

Although there are several alternatives in the state of the art to perform a wireless temperature sensor, we propose a new one based on the temperature-dependent capacitance of a liquid crystal cell. The combination of a simple RF antenna and the proposed sensor produces a LC circuit with a characteristic resonant frequency. As the capacitance of the LC cell changes with the temperature, the resonant frequency shifts with it. Then, a temperature monitoring can be carried out without physical connection or power supplies in the sensor circuit. In addition, the electric behaviour of the complete circuit has been analysed and modelled. In this sense, a simple and accurate electric equivalent circuit has been proposed. This allows a simple analysis of the sensing system by using equivalent discrete elements. This equivalent model has been tested by fabricating an experimental proof-of-concept. The agreement between experimental and simulated results is almost complete, successfully verifying the proposed model. The low cost of LC cells, the high sensitivity of LC-based temperature sensor and the correct operation of the wireless connectivity make this prototype useful in several potential applications like our target of a wearable temperature sensor for biomedical monitorization.
